# Long-Term Clinical and Immunological Impact of Severe COVID-19 on a Living Kidney Transplant Recipient – A Case Report

**DOI:** 10.3389/fimmu.2021.741765

**Published:** 2021-09-08

**Authors:** Liru Qiu, Ji Zhang, Yafei Huang, Gen Chen, Zhishui Chen, Changsheng Ming, Xia Lu, Nianqiao Gong

**Affiliations:** ^1^Department of Pediatrics, Tongji Hospital, Tongji Medical College, Huazhong University of Science and Technology, Wuhan, China; ^2^Department of Urology, The First Affiliated Hospital of Anhui Medical University, Hefei, China; ^3^Institute of Urology, Anhui Medical University, Hefei, China; ^4^Anhui Province Key Laboratory of Genitourinary Diseases, Anhui Medical University, Hefei, China; ^5^Institute of Organ Transplantation, Tongji Hospital, Tongji Medical College, Huazhong University of Science and Technology, Key Laboratory of Organ Transplantation of Ministry of Education, National Health Commission and Chinese Academy of Medical Sciences, Wuhan, China; ^6^Department of Immunology, Tongji Medical College, Huazhong University of Science and Technology, Wuhan, China; ^7^Department of Radiology, Tongji Hospital, Tongji Medical College, Huazhong University of Science and Technology, Wuhan, China

**Keywords:** COVID-19, living kidney transplant, lung injury, renal tubular injury, immunity

## Abstract

The long-term impact of COVID-19 on transplant recipients remains unknown. We describe the case of a 30-year-old male kidney transplant recipient from Wuhan, China that was treated for severe COVID-19 in February 2020. He suffered an acute lung and renal injury and required systemic treatment including adjustment of his immunosuppressant regime. He was followed up to 1-year after discharge. No chronic lung fibrosis or deterioration of his pulmonary function was observed. Despite COVID-19 mediated damage to his renal tubular cells, no transplant rejection occurred. His immunological profile demonstrated both cellular anti-SARS-CoV-2 reactivity and specific humoral immunity, indicating that it is beneficial for the transplanted patients to be immunized with SARS-CoV-2 virus vaccine. This case will help guide clinical decision making for immunocompromised individuals that become infected with SARS-CoV-2.

## Introduction

The Coronavirus Disease 2019 (COVID-19) has had an unprecedented impact on health systems worldwide, with over 160 million confirmed cases to date. However, the impact of severe acute respiratory syndrome coronavirus 2 (SARS-CoV-2) infection on solid organ transplant recipients remains largely unknown. While understanding of the early effects of the virus is emerging, longer term clinical and immunological outcomes in this immunosuppressed patient group have yet to be described. Understanding the longer-term effects of COVID-19 of transplant organ function and the immunological response of transplant recipients will help guide clinical decision making for this vulnerable patient group. This report describes the 1-year clinical and immunological outcomes of a living kidney transplant recipient from Wuhan, China who suffered severe COVID-19 in February 2020.

## Case Description

This case report describes a 30-year-old man who was diagnosed with end stage renal disease (ESRD) in September 2012 without a clear pathoetiology. He was initially treated with 6-months of hemodialysis before receiving a living kidney transplant from his mother in March 2013 using a recognized protocol (1, 2). The transplant kidney was from a confirmed cytomegalovirus negative patient, with no relevant comorbidities or infection history. After transplantation, he was initiated on an immunosuppressive regimen of tacrolimus, mycophenolic acid (MMF) and prednisone. He recovered well from surgery with no postoperative complications. He maintained stable kidney function (with a serum creatinine (Cr) level of approximately 138 μmol/L) with a tacrolimus trough level of 5 μg/L, MMF at 1g/day and prednisone at 2.5 mg/day as of January 7^th^ 2020.

**Figure 1:**
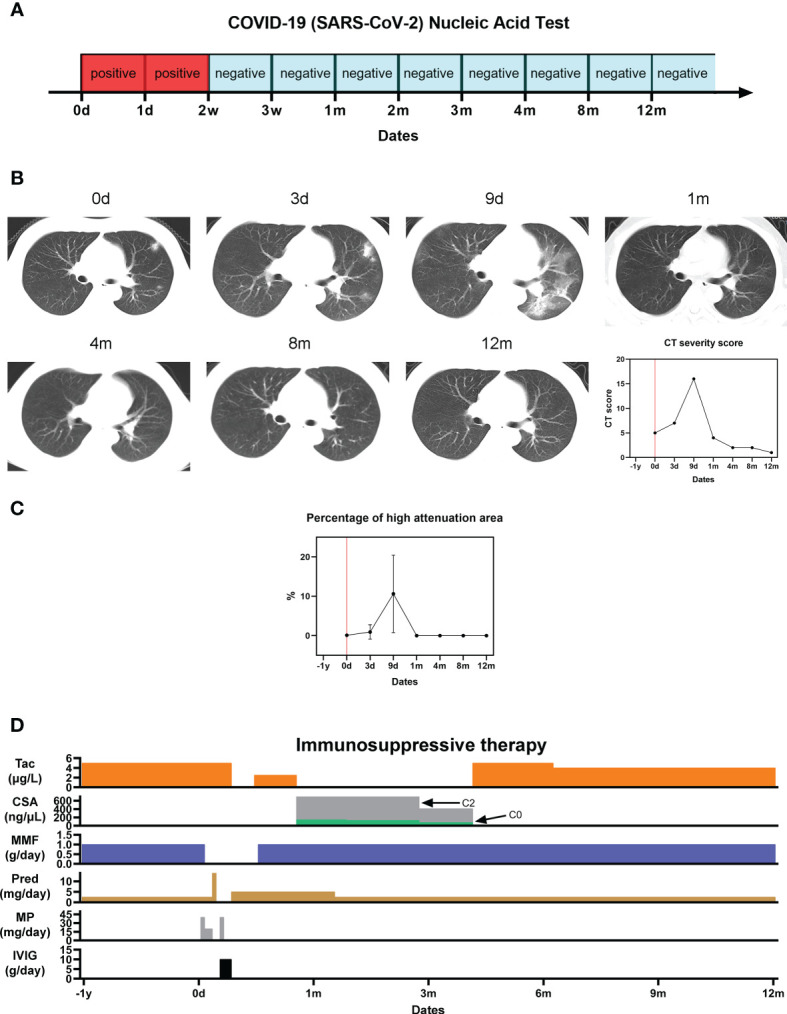
Pulmonary sequelae of COVID-19. **(A)** SARS-Cov-2 nucleic acid test results; **(B)** Serial CT thorax images at different timepoints from initial presentation of symptoms, and the corresponding COVID-19 CT severity score; **(C)** Percentage of high attenuation area (HAA%) on CT imaging indicating pulmonary fibrosis; **(D)** Immunosuppressive therapy regime before, during and after the patient’s hospitalization for severe COVID-19.

On January 31^st^ 2020 (date of first onset, D0), he became pyrexial (39°C), with myalgia, anorexia and a tachycardia of 120 bpm. A nasopharyngeal swab specimen obtained and SARS-CoV-2 ribonucleic acid (RNA) was detected using conventional qualitative reverse transcription polymerase chain reaction (**Figure 1A**). The SARS‐CoV‐2 specific primers used were as follows: forward primer 5′‐ACTTCTTTTT CTTGCTTTCGTGGT‐3′; reverse primer 5′‐GCAGCAGTACGCACACAATC‐3′; and the probe 5′CY5‐CTAGTTACACTAGCCATCCTTACTGC‐3′BHQ1. A computed tomography (CT) scan of the thorax demonstrated extensive ground glass opacity across the left lung field (**Figures 1B, C**), and routine blood tests revealed a lymphocytopenia. A diagnosis of COVID-19 was made.

He clinically deteriorated and received supportive therapy with oseltamivir, antibiotics, and supplementary oxygen through a non-rebreather mask. His immunosuppressant regime was adjusted in response to his acute illness. After escalation of his oral prednisone over the first 2-days (2.5 mg/day), methylprednisolone was given intravenously from D2 to D4 (40 mg/day on D2, 20 mg/day on D3 and D4) due to worsening clinical symptoms and lung CT which demonstrated pulmonary oedema consistent with severe COVID-19. MMF was withdrawn from D3. Intravenous immunoglobulin (IVIG) was given from D7 to D9 (10 g/day). In response to his worsening clinical status, his tacrolimus was withdrawn from D10 to D15, before being re-initiated at half its normal dose (2.5 μg/L). On D27 after symptom onset, his tacrolimus was converted to Cyclosporin A (CsA, C2 = 799.6 ng/μL, C0 = 144.7 ng/μL). By D26, his symptoms had completely resolved, his lung CT had improved, and his nasopharyngeal specimen was negative for SARS-CoV-2 (3). He was deemed suitable for discharge. His normal 6mmunosuppression regime was re-established by 4-months after symptom onset (5 μg/L tacrolimus, 1 g/day MMF and 2.5 mg/day prednisone) (**Figure 1D**).

The patient’s clinical and immunological response to severe COVID-19 was followed up to 1-year from symptom onset.

### Serial SARS-Cov-2 Ribonucleic Acid Testing

The patient’s nasopharyngeal swab test specimen remained positive for SARS-Cov-2 RNA for 2 weeks following symptom onset before being tested negative at every subsequent interval during the one-year follow-up window (**Figure 1A**).

### Lung Function and Fibrosis

His lung CT revealed patchy shadows and ground-glass opacities within 9 days after the onset of the disease, and then gradually improved, and completely returned to normal at 1 month. A semi-quantitative CT severity scoring of COVID-19 first proposed by Marco et al. (4, 5) was calculated for each of the five pulmonary lobes considering the extent of anatomic involvement, as follows: 0, no involvement; 1, < 5% involvement; 2, 5–25% involvement; 3, 26–50% involvement; 4, 51–75% involvement; and 5, > 75% involvement. The resulting global CT score was the sum of the individual lobar scores (i.e., between 0 and 25). The CT score was consistent with the trend of the imaging findings. For the quantitative CT evaluation of pulmonary fibrosis, corresponding slices were selected from the CT images in the regions affected by COVID-19 in each lobe. The extent of fibrosis was determined by calculating the percentage of each lobe with a high attenuation area (HAA%). HAA was defined by a attenuation value between -600 and -250 Hounsfield Units (6). The percentage of high attenuation area (HAA%) approached 0% at 1 month after onset and remained 0% at 1-year, suggesting no pulmonary fibrosis following the acute COVID-19 episode (**Figures 1B, C**).

### Renal Function

**Figure 2:**
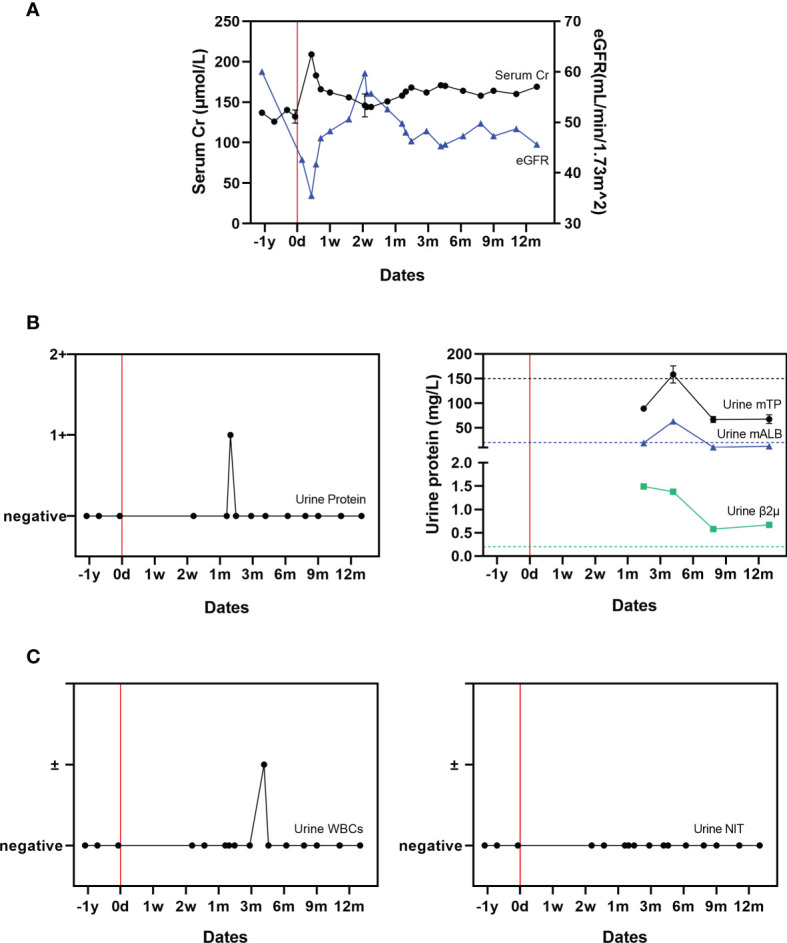
Changes in renal function related to COVID-19. **(A)** Serial serum creatinine and eGFR measurements; **(B)** Urine protein, micro total protein, micro albumin, and β2 microglobulin measurements. **(C)** Urinary WBCs and urinary nitrite (NIT) measurements. The mean value derived from two uninfected paired controls is shown using dotted lines as a comparator.

The patient’s kidney function was transiently impaired shortly after onset of severe COVID-19 (**Figures 2A, B**). On D3, his serum Cr level increased to 209 μmol/L while estimated glomerular filtration rate (eGFR) decreased to 35.5 ml/min/1.73m^2^. Following this, his kidney function gradually recovered. By month 12, his serum Cr level was 169 μmol/L, while eGFR had returned to 45.60 ml/min/1.73m^2^. Notably, his urine protein level reached 1+ (0.2-10 g/L) transiently on D49, but remained negative afterward. His urine micro total protein (mTP) increased during month 2 to 89.0 mg/L, gradually increased to its peak as 158.50 mg/L during month 4, and had decreased back to 67.50 mg/L by month 12. The quantity of urine microalbumin (mALB) was 19.00 mg/L at month 2, highest during month 4 at 63.00 mg/L, and had returned to 12.60 mg/L by month 12. Urine β2 microglobulin (β2μ) decreased from 1.49 mg/L at month 2 to 0.67 mg/L by month 12. His urinary WBC level briefly reached suspicious positive on D125, and was otherwise negative. His urinary nitrite (NIT) levels always remained negative (**Figure 2C**). During the acute disease phase, the patient’s urine output remained stable (1500-2000 mL/day).

In order to make comparison to a non-infected control, serial testing and follow-up of two most recent similar living kidney transplant recipients, of the same gender and with similar baseline characteristics (e.g., age, BMI, donor history, immunosuppression regime) with stable kidney function and without a rejection episode was performed. A comparison to the paired controls is presented in **Supplementary Table 1**.

### Cellular and Humoral Immunity

**Figure 3:**
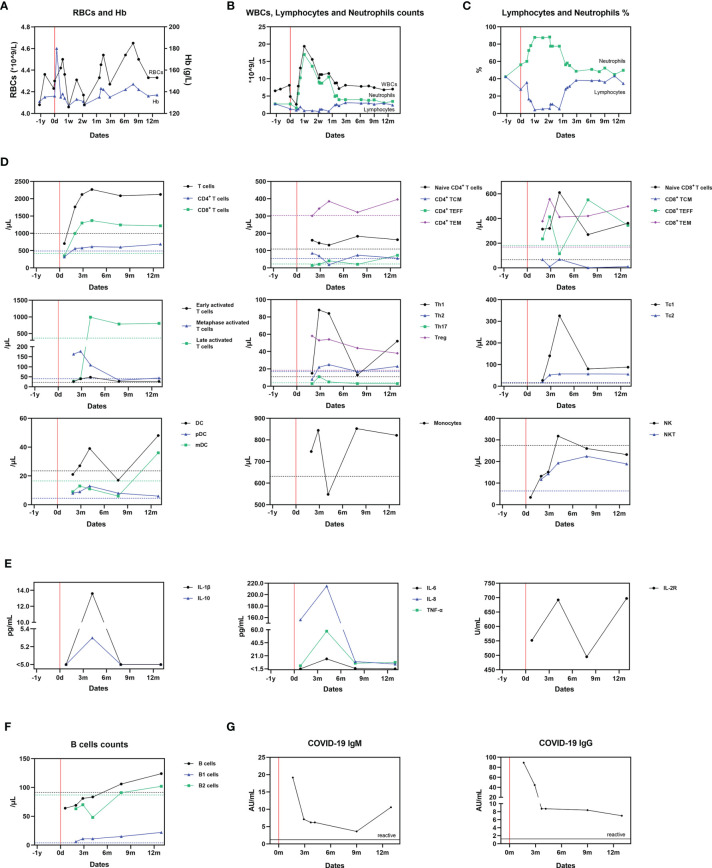
Dynamic changes of cellular and humoral immunity after SARS-CoV-2 infection. **(A)** Serum RBCs and Hb measurements; **(B)** Serum WBCs, lymphocyte and neutrophil counts; **(C)** Serum lymphocytes and neutrophils proportions; **(D)** The proportion of immunocytes; **(E)** Cytokines levels; **(F)** B cell counts; **(G)** Antibodies against SARS-Cov-2. The mean value derived from two uninfected paired controls is shown using dotted lines as a comparator in **(A–E)**.

The patient’s red blood cell count (RBC) and serum hemoglobin (Hb) stayed within normal range throughout the acute COVID-19 infection and follow-up period (**Figure 3A**). An elevated white blood cell (WBC) count, neutrophillia, and lymphocytopenia (**Figures 3B, C**) were identified at the onset of symptoms. This had gradually returned to baseline by month 2; this is consistent with the current understanding of changes in white cell count and proportions during acute SARS-CoV-2 infection (7).

Alterations in immunocytes were detected by flow cytometry following antibody staining of their surface markers at Wuhan KingMed Diagnostics (Wuhan, China). To monitor the impact of COVID-19 on the immune system of the recipient, serum samples containing peripheral blood mononuclear cells (PBMCs) were collected at month 1, 2, 3, 4, 8 and 13. At month 1, the levels of T cells, CD4+ T cells, CD8+ T cells, B cells and NK cells were lower compared with the controls, which could be explained by the cytolytic effect during the acute progressive phase of COVID-19 (**Figures 3D–F**). Afterward, compared to the uninfected paired controls, T-cell (CD3^+^)/CD8^+^ T-cell (CD3^+^, CD8^+^) counts of the infected recipient increased. Metaphase (CD3^+^, CD25^+^) and late activated T-cells (CD3^+^, HLADR^+^), Th1-cells (IFN-γ^+^, CD4^+^) and T-regulatory cells (CD3^+^, CD4^+^, CD25^high^, CD127^dim^) also increased, whilst Th2 (IL-4^+^, CD4^+^) and Th17 (IL-17^+^, CD4^+^) levels remained stable. The levels of circulating CD4^+^, naive T-cells (CD3^+^, CD4^+^, CCR7^+^, CD45RA^+^), central memory T-cells (TCM; CD3^+^, CD4^+^, CCR7^+^, CD45RA^-^), effector T-cells (TEFF; CD3^+^, CD4^+^, CCR7^-^, CD45RA^+^) and effector memory T-cells (TEM; CD3^+^, CD4^+^, CCR7^-^, CD45RA^-^) fluctuated around the controls with no clear patterns. Regarding CD8^+^ cells, the level of Tc cells increased, particularly Tc1 cells (IFN-γ^+^, CD8^+^). Naïve T-cells (CD3^+^, CD8^+^, CCR7^+^, CD45RA^+^), TCM (CD3^+^, CD8^+^, CCR7^+^, CD45RA^-^) and TEM (CD3^+^, CD8^+^, CCR7^-^, CD45RA^-^) also markedly increased. Dendritic cell (DC; Lin^-^, HLA-DR^+^) levels remained similar to the paired controls, however, at month 13, the levels of DC and myeloid DC (mDC; Lin^-^, HLA-DR^+^, CD11c^+^) both increased sharply. The serum levels of monocytes and NK (CD3^-^, CD56^+^) cells fluctuated, whilst NKT (CD3^+^, CD56^+^) levels increased throughout the follow-up period in the infected patient (**Figure 3D**, **Supplementary Figure 1**).

Sera were collected to measure cytokine concentration at month 1, 4, 8 and 13 by the clinical laboratory of Tongji Hospital (Wuhan, China). The concentration of IL-1β, IL-2R, IL-6, IL-8, IL-10 and TNF-α increased from month 1 to 4, and decreased after month 4. Only the concentration of IL-2R rose again after month 7, but also remained at normal level (233-710U/mL) (**Figure 3E**).

At month 1, the B-cell (CD19+, CD3-) count of the recipient was lower than the controls. However, after this, it gradually increased to a slightly higher level than the controls (**Figure 3F**). B1-cell (CD19^+^, CD3^-^, CD5^+^) counts of the recipient remained higher than in the controls for the duration of follow-up (month 2 to 13). The IgM and IgG antibodies against SARS‐CoV‐2 in serum specimens were detected using YHLO‐CLIA‐IgG and YHLO‐CLIA‐IgM kits supplied by YHLO (YHLO Biotech Co. Ltd Shenzhen, China) and interpreted with ≥ 1.2 AU/mL indicating as a reactive (positive) test and < 1.2 AU/mL as a nonreactive (negative) test. The IgM antibody levels were high in the infected patient during month 2 (>1.2 AU/mL) before gradually decreasing up to month 9. After month 9 and then increased to a relatively high level. The IgG antibody achieved its peak level at month and then decreased but still remained positive throughout the follow-up window (**Figure 3G**).

## Discussion

This report describes the case of a young kidney transplant recipient who was diagnosed with severe COVID-19. He had a typical clinical timeline from onset of symptoms, to worsening hypoxia requiring oxygen supplementation, viral clearance, and eventually rehabilitation. Although his nasopharyngeal swab specimen was negative two weeks after initial detection (indicating clearance of the SARS-CoV-2 virus) and the recipient had recovered symptomatically after one month, his severe episode of COVID-19 in the presence of immunocompromise had chronic physiological and immunological effects.

During the acute progressive period of COVID-19, whilst the patient was receiving intravenous methylprednisolone in response to his worsening pulmonary oedema, MMF withdrawal was followed by tacrolimus reduction and withdrawal. After his clinical situation had improved, CsA was initiated for 3-month before re-initiation of tacrolimus. CsA administration was considered as we hypothesized that this may directly inhibit viral proliferation (8, 9). Conversion back to tacrolimus was performed with close monitoring for calcineurin inhibitor (CNI) toxicity. Finally, MMF was re-initiated once his symptoms had fully resolved. Interestingly, the patient’s immunocompromised status may be beneficial in avoiding the severe ‘cytokine storm’ which increases risk of mortality from COVID-19 (10, 11). However, minimizing or withdrawing immunosuppression is often necessary during severe viral interstitial inflammation of the lung. Carefully balancing these potential risks and benefits is essential for transplant recipients that develop COVID-19; further data is needed to inform clinical decision making in the future (12–14).

In this case, a CT thorax scan post resolution of symptoms demonstrated that the acute lung damage initially observed had fully resolved. The long-term effects of COVID-19 on pulmonary function remain relatively unexplored (15). By the completion of 1-year follow-up, no chronic fibrosis was detected on imaging in this kidney transplant recipient.

The patient’s kidney function was impaired during the acute progressive phase of COVID-19 with a markedly increased serum Cr level and decreased eGFR. Though his Cr level and eGFR eventually recovered to the normal level, the mechanism by which the SARS-CoV-2 virus impairs renal function remains unknown (16, 17). Before his acute COVID-19 episode, he had never previously had detectable proteinuria in the 7-years since his living donor transplant. Interestingly in this case, proteinuria occurred for the first time after he had cleared the SARS-CoV-2 virus and recovered from symptomatic COVID-19; urinary protein rose transiently on D40 and urine β2 microglobulin between month 2 and 12. This suggests a potential mechanism of chronic damage to renal tubular cells mediated by the SARS-CoV-2 virus. Urinary WBC and NIT levels largely remained negative throughout, which suggest that the proteinuria observed was not caused by a urinary tract infection.

SARS-CoV-2 exhibited a cytolytic effect during the acute progressive phase of COVID-19, as demonstrated by the patient’s decreased lymphocyte counts, especially T cells, CD4^+^ T cells, CD8^+^ T cells, B cells and NK cells. Regarding his immunoreactivity, firstly we compared alloimmunity after SARS-CoV-2 infection over the follow-up window between month 3 to 12. DC levels remained similar to the paired controls, whilst circulating CD4+, naive T-cells, central memory T-cells, effector T-cells and effector memory T-cells fluctuated around the controls. Considering no clinical rejection episode occurred despite changes to the intensity of immunosuppression, we can deduce that SARS-CoV-2 infection had no impact on immunoreactivity against alloantigens in this recipient. However, the risk of rejection has been reported to be increased after SARS-CoV-2 infection (18), and this requires further investigation in larger, multi-center studies. With regard to evaluate general immune status after SARS-CoV-2 infection, *ex vivo* T cell response to polyclonal T cell stimulation may be helpful. A recent report showed that no difference was identified between transplanted recipients and immunocompetent individuals (19). Therefore, more investigations are required to elucidate the general immune status in patients after convalescence.

We detected persistent immunoreactivity against the virus during the follow-up window. From month 1 to month 4, late-activated T-cells and IFN-γ^+^ CD4^+^ Th1 cells markedly increased in the infected patient, indicating lasting antiviral Th-cell activation. The increase of CD8^+^ T cells, IFN-γ^+^ CD8^+^Tc1, CD8^+^TEFF, and CD8^+^TEM also indicates antiviral Tc activation. After month 4, the counts of these cells decreased or stabilized. We further detected the expression levels of cytokines which reflect the functioning of the immune cells. IL-1β, IL-2R, IL-6, IL-8, IL-10 and TNF-α gradually increased within 4 months, and gradually decreased after 4 months, which were similar to the change the above-mentioned trend of T cells. Briefly, SARS-CoV-2-reactive functional T cell responses increased after infection and decreased slowly afterward. DC, pDC and monocyte levels remained marginally higher in the infected patients than controls throughout the follow-up. Myeloid dendritic cells were detected during month 3 to 9, after the acute phase. This indicates a potential long-term effect of DC against the virus. The detection of raised levels of monocytes, NK cells and NKT cells in this case suggests intact innate immunity after SARS-CoV-2 virus infection in this patient.

We performed an analysis of this transplant recipient’s humoral immunity, including specific antibodies against SARS-CoV-2 virus, in order to evaluate the potential protective effect of infection against future re-infection. From month 2 to 4, B-cell and B2-cell levels were lower than controls. However, after this initial phase, the patient’s B- and B2-cell levels increased whilst his B1-cells were consistently detected at higher levels than controls. The anti-SARS-CoV-2 IgM decreased from its early peak up to month 9, but remained within a titer range that was considered protective, then subsequently rose again. It is unlikely that this event signified re-infection or re-exposure for the following reasons: (1) serial nasopharyngeal swab testing for SARS-CoV-2 RNA remained negative until month 12; (2) the patient had no clinical symptoms suggestive of re-infection; (3) His IgG titer continually decreased until M12; (4) the changes observed in levels of DCs, mDCs and B-cells showed a trend consistent with IgM levels. A very recent study shows that SARS-CoV-2 infection induces a robust antigen-specific, long-lived humoral immune response in humans (20). We therefore believe that this transplant recipient exhibited immunoprotection against SARS-CoV-2 virus, with effective titer of specific antiviral IgM and IgG (21). These data suggest that transplant recipients may display sufficient immunoreactivity to generate protective antibodies against SARS-CoV-2, which supports expansion of vaccination programmes to include transplant recipients. This may also have implications for other immunocompromised populations.

In summary, this living kidney transplant recipient suffered an episode of severe COVID-19 consistent with current understanding of the disease phenotype. He recovered clinically within one month of infection, and nasopharyngeal swab testing demonstrated clearance of the SARS-CoV-2 virus. While COVID-19 appeared to have no chronic impact on the lung function or fibrosis, it may have resulted in lasting damage to renal tubular cells. Over a 1-year follow-up window, the patient demonstrated both an effective cellular and specifical humoral anti-SARS-CoV-2 response, demonstrating that it is beneficial for the transplanted patients to be immunized with SARS-CoV-2 virus vaccine. This experience provides novel insights into the long-term effects of the SARS-CoV-2 virus, and can inform clinical decision making for immunocompromised individuals that become infected.

## Data Availability Statement

The raw data supporting the conclusions of this article will be made available by the authors, without undue reservation.

## Ethics Statement

Written informed consent was obtained from the individual(s) for the publication of any potentially identifiable images or data included in this article.

## Author Contributions

JZ, YH, GC, XL and NG contributed to the conception of the study. JZ and YH performed the experiment. LQ, JZ, YH, XL and NG contributed significantly to analysis and manuscript preparation. LQ, JZ, ZC, CM, XL and NG helped perform the analysis with constructive discussions. All authors contributed to the article and approved the submitted version.

## Funding

This work was supported by grants to NG from the National Natural Science Foundation of China (No. 81570678), to NG from the Major State Basic Research Development Program of China (NO. 2013CB530803, 973), to XC from the Special Project of the Ministry of Health (201302009), and to NG from the Clinical Research Physician Program of Tongji Medical College, HUST.

## Conflict of Interest

The authors declare that the research was conducted in the absence of any commercial or financial relationships that could be construed as a potential conflict of interest.

## Publisher’s Note

All claims expressed in this article are solely those of the authors and do not necessarily represent those of their affiliated organizations, or those of the publisher, the editors and the reviewers. Any product that may be evaluated in this article, or claim that may be made by its manufacturer, is not guaranteed or endorsed by the publisher.
