# Supplementary material for: Long-Term Clinical and Immunological Impact of Severe COVID-19 on a Living Kidney Transplant Recipient – A Case Report
Source: Front Immunol. 2021 Sep 8;12:741765. doi: 10.3389/fimmu.2021.741765 (PMC8456079; doi:10.3389/fimmu.2021.741765)
Supplement: Supplementary file 1 [file DataSheet_1.docx]

Supplementary Table 1. Demographic (the recipient, and two controls)

|  | The recipient | Control-1 | Control-2 |
| --- | --- | --- | --- |
| Sex  Age (years)  BMI  Donor background  Donor age (years)  Donor gender  Kidney GFR before donation  Immunological background  Panel reactive antibody (%)  MHC-I  MHC-II  Mismatch of six HLA antigens  Surgery background  Pretransplant dialysis type  Prior dialysis time（mon）  Warm ischemia time (minutes)  Cold ischemia time (minutes)  Immunosuppressive regime  Time post transplantation (years) | Male  23  18.4  47  Female  38.94  2.5  1.9  0  Hemodialysis  3  155  2  Tac+MMF+prednisone  7 | Male  22  17.8  44  Female  42.14  0  0  1  Hemodialysis  3  180  1  Tac+MMF+prednisone  7 | Male  25  21.0  49  Female  46.19  5.7  2  0  Hemodialysis  3  120  1  Tac+MMF+prednisone  6 |

MHC: major histocompatibility complex；

GFR: glomerular filtration rate (detected before donation)

Supplementary Figure 1.

Dynamic Changes of the counts of immunocytes after COVID-19 onset

|  |  |
| --- | --- |
|  |  |
|  |  |
|  |  |
|  |  |
|  |  |
